# Supplementary material for: Dynamic transcriptomic profiles of zebrafish gills in response to zinc supplementation
Source: BMC Genomics. 2010 Oct 11;11:553. doi: 10.1186/1471-2164-11-553 (PMC3091702; doi:10.1186/1471-2164-11-553)
Supplement: Additional file 2 — Interactive Direct Interaction Network representing the molecular interactions between zinc, copper, iron, calcium and proteins encoded by transcripts changed by zinc supplementation. Mini web-site containing index.html and hyperlinked pages in subdirectory describing a Direct Interaction Network automatically generated based on curated interactions contained within the proprietary PathwayArchitect database. Ovals represent proteins and the circles symbolize metal ions. Objects are coloured by their abundance in zebrafish at the time-point they were significantly different from the control is a scale from -4 fold (dark green) to +4 fold (dark red). Where significant differences were found at more than one time-point, the colour overlay shows expression at the first instance. Dark blue squares denote 'binding', and light blue squares 'expression'; green squares stand for 'regulation', green diamonds for 'metabolism', and green circles for 'promoter binding'. Arrow heads indicate directionality of the interaction where annotated. All nodes and edges can be further interrogated by selecting the relative area of the image. [file 1471-2164-11-553-S2.zip › PathwayArchitect Zn xs DIN/78720.html]

# PROTEIN: DAB2

|  |  |
| --- | --- |
| Name | DAB2 |
| Type | PROTEIN |
| Description | disabled homolog 2, mitogen-responsive phosphoprotein (Drosophila) |
| Note | DAB2 mRNA is expressed in normal ovarian epithelial cells but is down-regulated or absent from ovarian carcinoma cell lines. The 770-amino acid predicted protein has an overall 83% identity with the mouse p96 protein, a putative mitogen-responsive phosphoprotein; homology is strongest in the amino-terminal end of the protein in a region corresponding to the phosphotyrosine interaction domain. The down-regulation of DAB2 may play an important role in ovarian carcinogenesis. This gene was initially named DOC2 (for Differentially expressed in Ovarian Cancer) and is distinct from the DOC2A and DOC2B genes (for double C2-like domains, alpha and beta). |
| Alias | disabled homolog 2, mitogen-responsive phosphoprotein (Drosophila) |
|  | disabled (Drosophila) homolog 2 (mitogen-responsive phosphoprotein) |
|  | AI957090 |
|  | Doc2 |
|  | disabled homolog 2 mitogen-responsive phosphoprotein (Drosophila) |
|  | p96 |
|  | disabled homolog 2 |
|  | Dab2 |
|  | DOC-2 p82 isoform |
|  | AA960054 |
|  | D15Wsu122e |
|  | Mitogen-responsive phosphoprotein |
|  | 5730435J12Rik |
|  | mitogen-responsive phosphoprotein |
|  | DAB2 |
|  | DOC2 |
|  | Differentially expressed protein 2 |
|  | DOC-2 |
|  | Doc-2 |


---

|  |  |
| --- | --- |
| GO ID | GO:0007399 |
|  | GO:0030308 |
|  | GO:0005515 |
|  | GO:0008283 |
|  | GO:0007275 |
|  | GO:0006898 |
|  | GO:0007588 |
|  | GO:0001701 |
|  | GO:0000904 |


---

|  |  |
| --- | --- |
| MIM | MIM:601236 |


---

|  |  |
| --- | --- |
| Connectivity | 141 |


---

|  |  |
| --- | --- |
| Entrez ID | 13132 |
|  | 1601 |
|  | 79128 |


---

|  |  |
| --- | --- |
| Agilent ID | A\_53\_P177536 |
|  | A\_44\_P513385 |
|  | A\_53\_P151208 |
|  | A\_43\_P12412 |
|  | A\_23\_P257871 |
|  | A\_52\_P17534 |
|  | A\_51\_P157994 |
|  | A\_14\_P125479 |


---

|  |  |
| --- | --- |
| Pathway | Zn xs inventory |
|  | Zn xs DIN |


---

|  |  |
| --- | --- |
| GO Process | receptor mediated endocytosis |
|  | cell proliferation |
|  | negative regulation of cell growth |
|  | cellular morphogenesis during differentiation |
|  | neurogenesis |
|  | embryonic development (sensu Mammalia) |
|  | development |
|  | excretion |


---

|  |  |
| --- | --- |
| UniGene | Hs.481980 |
|  | Rn.14763 |
|  | Mm.240830 |


---

|  |  |
| --- | --- |
| Affymetrix Probeset ID | 104633\_at |
|  | 1368202\_a\_at |
|  | 1372031\_at |
|  | 1420498\_a\_at |
|  | 1423805\_at |
|  | 1429693\_at |
|  | 1430604\_a\_at |
|  | 165792\_at |
|  | 201278\_at |
|  | 201279\_s\_at |
|  | 201280\_s\_at |
|  | 210757\_x\_at |
|  | 232898\_at |
|  | 240873\_x\_at |
|  | 42819\_at |
|  | 45341\_at |
|  | 479\_at |
|  | 66805\_at |
|  | 82122\_at |
|  | 98044\_at |
|  | 98045\_s\_at |
|  | aa407284\_rc\_s\_at |
|  | g13111753\_3p\_a\_at |
|  | g4503250\_3p\_a\_at |
|  | 112694\_at |
|  | 112693\_at |
|  | Hs.128807.0.S1\_3p\_at |
|  | Hs.292724.0.A1\_3p\_at |
|  | Hs.81988.0.S3\_3p\_at |
|  | rc\_AI233743\_at |
|  | u18869\_s\_at |
|  | U53446\_at |
|  | U95178\_s\_at |
|  | 1559370\_at |
|  | 130497\_at |
|  | 51367\_at |
|  | 52187\_at |
|  | 56015\_at |
|  | Hs2.364642.1.S1\_3p\_at |
|  | RC\_AA400292\_at |
|  | RC\_AA478971\_r\_at |
|  | RC\_AA478971\_s\_at |
|  | RC\_R62907\_at |
|  | TC17338\_at |
|  | TC31080\_s\_at |
|  | TC31081\_at |


---

|  |  |
| --- | --- |
| GO Function | protein binding |


---

|  |  |
| --- | --- |
| Nucleotide | AK017619 |
|  | AF045660 |
|  | AK154716 |
|  | U53446 |
|  | AK165810 |
|  | AK165519 |
|  | NM\_024159 |
|  | AF045657 |
|  | NM\_001008702 |
|  | AK162851 |
|  | U95178 |
|  | AF220562 |
|  | BC097314 |
|  | AF205890 |
|  | AF260580 |
|  | NM\_023118 |
|  | BC003064 |
|  | U41111 |
|  | AF045659 |
|  | AF260579 |
|  | U18869 |
|  | AF045658 |
|  | AK168375 |
|  | L16886 |
|  | U39050 |
|  | BC006588 |
|  | AK024965 |
|  | U95177 |
|  | BC016887 |
|  | AK002850 |
|  | AK162744 |
|  | AF188298 |
|  | NM\_001343 |


---

|  |  |
| --- | --- |
| Protein | BAE37084 |
|  | AAB19032 |
|  | AAC03360 |
|  | AAA98975 |
|  | NP\_077073 |
|  | BAE32784 |
|  | AAH06588 |
|  | AAF05540 |
|  | AAC33405 |
|  | BAE38234 |
|  | P98078 |
|  | NP\_001008702 |
|  | P98082 |
|  | AAH03064 |
|  | AAG44669 |
|  | NP\_001334 |
|  | NP\_075607 |
|  | AAH97314 |
|  | O88797 |
|  | BAB22405 |
|  | AAB02646 |
|  | AAF23161 |
|  | AAC03363 |
|  | AAC03362 |
|  | BAE38389 |
|  | BAE37047 |
|  | AAC50824 |
|  | AAH16887 |
|  | AAA93195 |
|  | AAC33406 |
|  | AAC03361 |


---

|  |  |
| --- | --- |
| Organism | Mammal |


---

|  |  |
| --- | --- |
| Location | chromosome 2, 2q16 (Rattus norvegicus) |
|  | chromosome 5, 5p13 (Homo sapiens) |
|  | chromosome 15, 15 6.7 cM (Mus musculus) |


---

|  |  |
| --- | --- |
